# Supplementary figures and images for: Montastraea cavernosa corallite structure demonstrates distinct morphotypes across shallow and mesophotic depth zones in the Gulf of Mexico
Source: PLoS One. 2019 Mar 26;14(3):e0203732. doi: 10.1371/journal.pone.0203732 (PMC6435134; doi:10.1371/journal.pone.0203732)

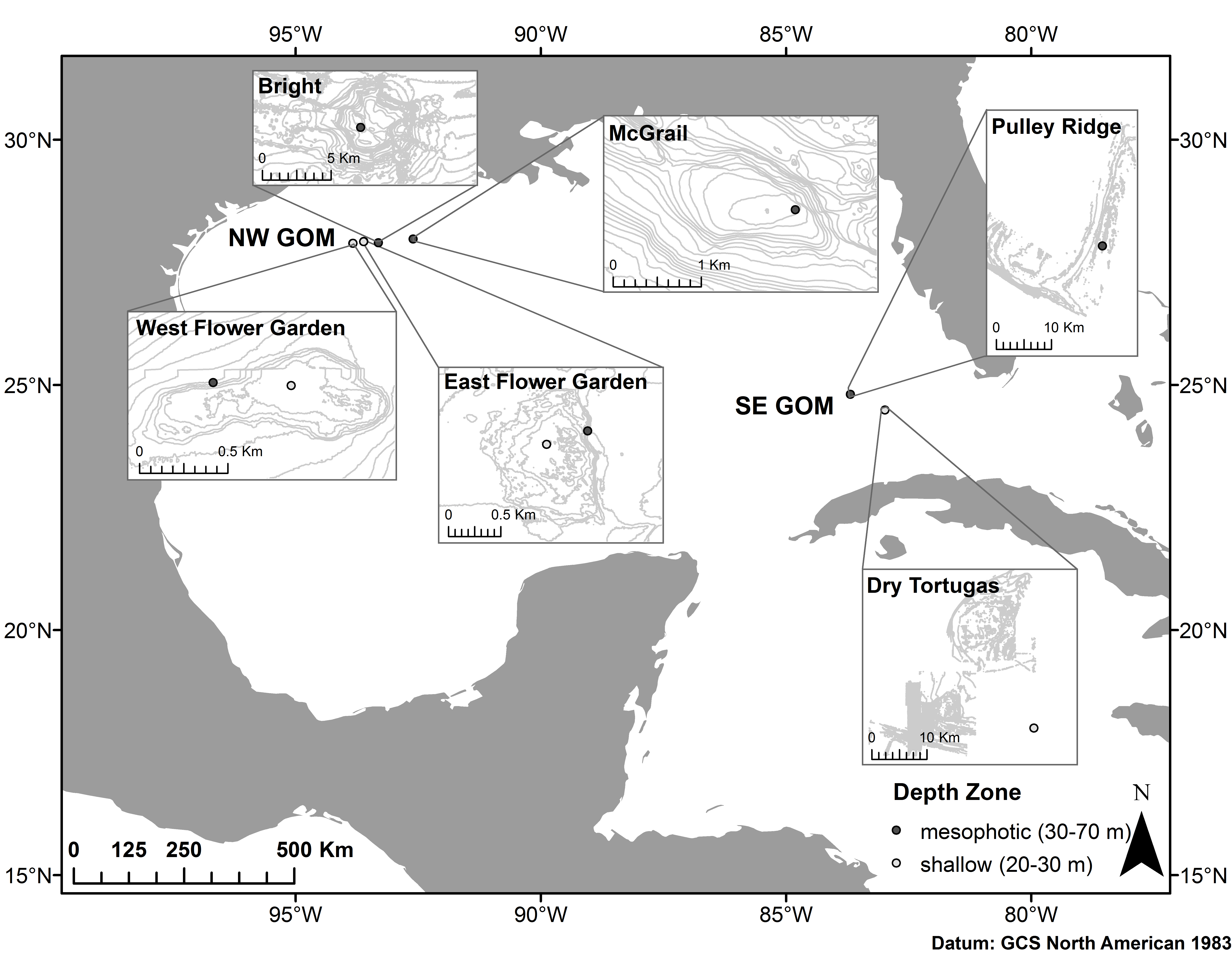

Supplement: S1 Fig — Map of the Gulf of Mexico, with inset boxes of six sampling sites in the northwest and southeast Gulf of Mexico (NW GOM and SE GOM, respectively). Inset overlays include available bathymetry data of sites, and locations of specimen collection color-coded by depth zone (mesophotic 30–70 m, shallow 20–30 m). Geographic coordinates as in Table 2. (TIF) [file pone.0203732.s001.tif]

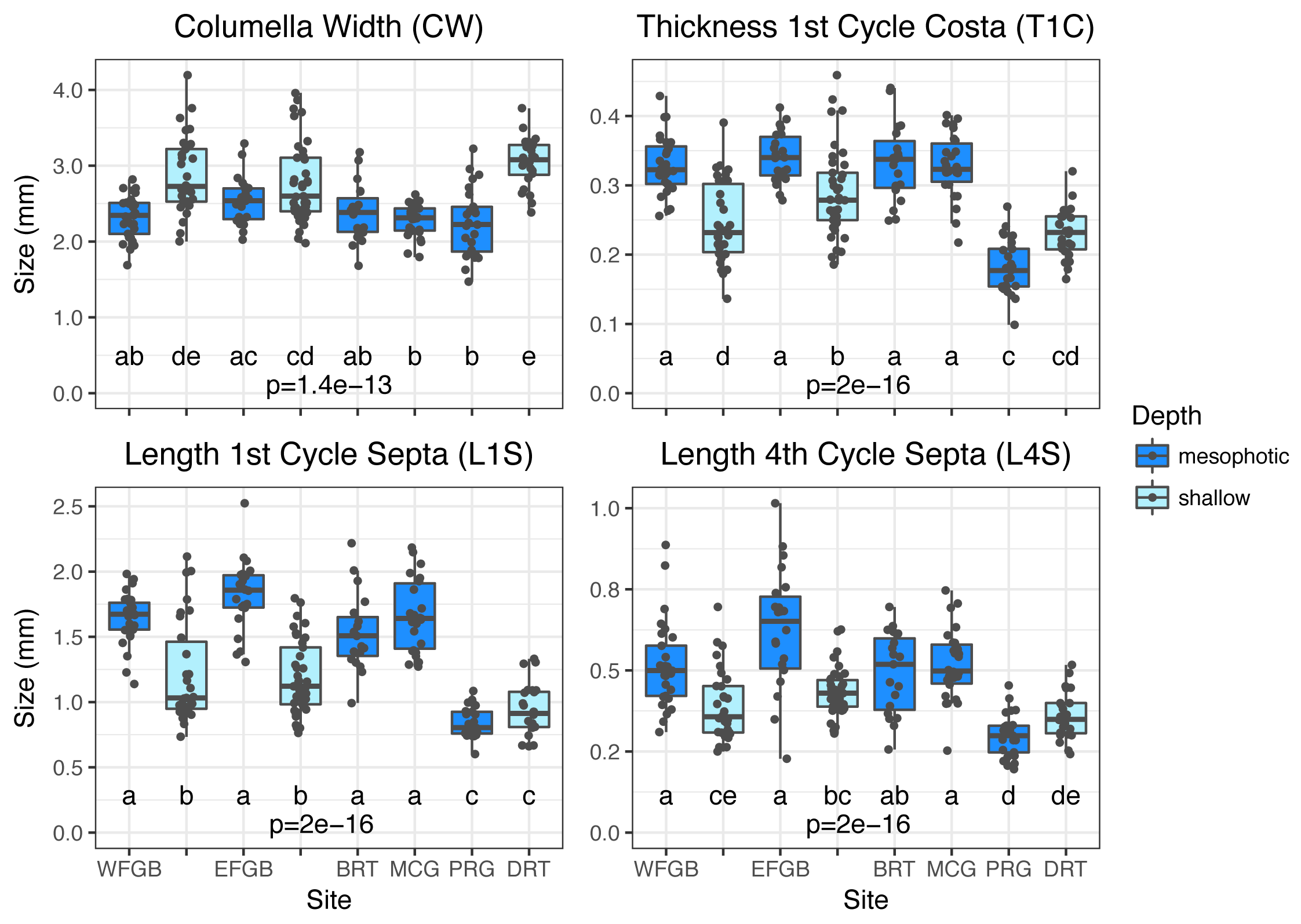

Supplement: S2 Fig — Boxplots with sample overlays for columella width (CW), thickness of the first cycle costa (T1C), length of the first cycle septa (L1S), and length of the fourth cycle septa (L4S) across six sites and two depth zones in the Gulf of Mexico. Overall p values represent Kruskal-Wallis tests across sites and depth zones for each metric and different letters denote significant differences (p<0.05) between pairwise comparisons of sites and depth zones generated by Dunn’s tests. (TIF) [file pone.0203732.s002.tif]

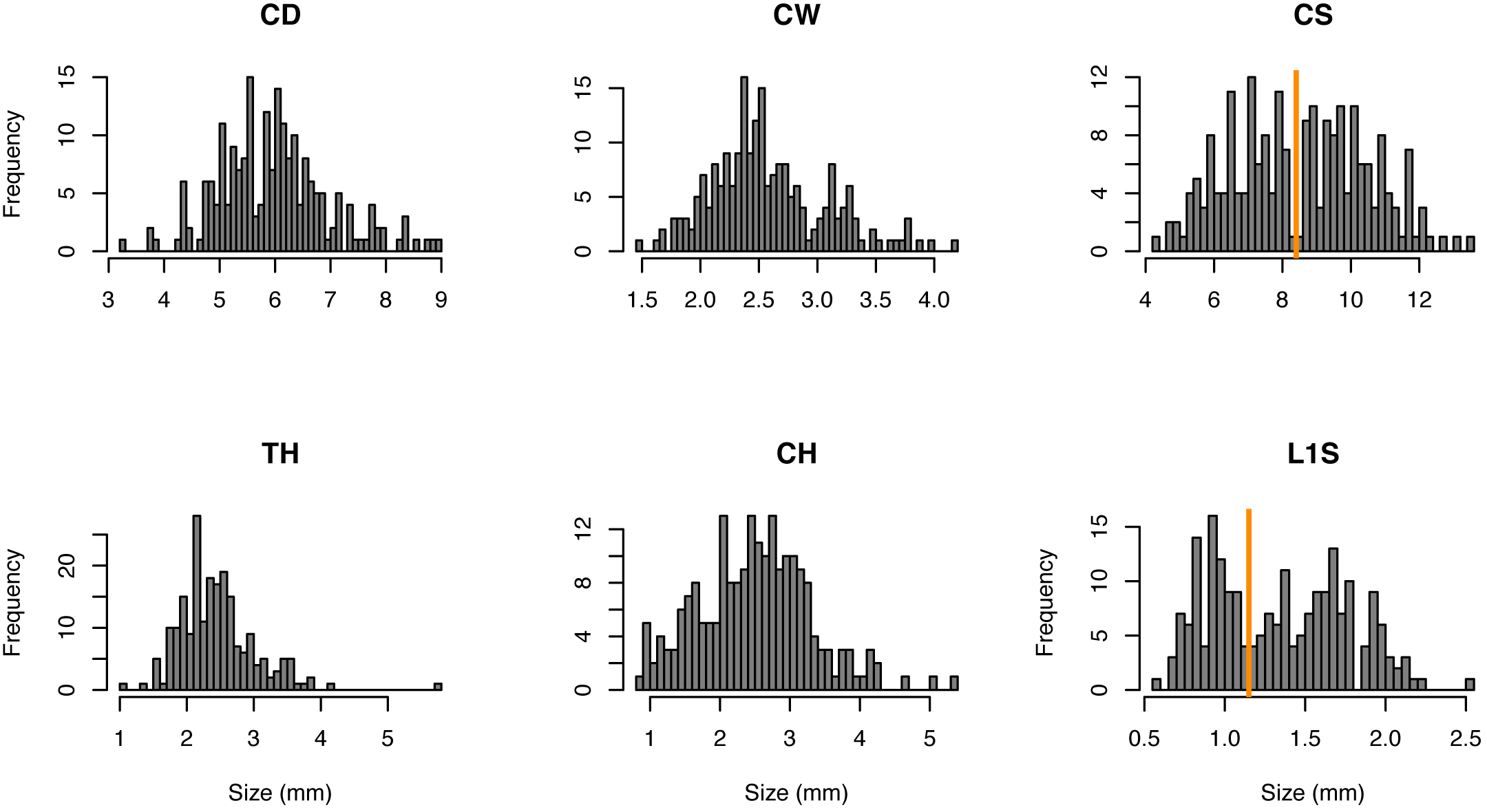

Supplement: S3 Fig — Frequency distributions for the six morphological metrics representing the majority of corallite variation across depth, including: corallite diameter (CD), columella width (CW), corallite spacing (CS), theca height (TH), corallite height (CH), and length of first cycle septa (L1S). The size threshold of CS (8.40 mm) is represented in the orange vertical line, denoting two morphotypes distinguished primarily by differences in corallite spacing. L1S also had a split distribution but had a less obvious threshold (1.15 mm). (TIF) [file pone.0203732.s003.tif]

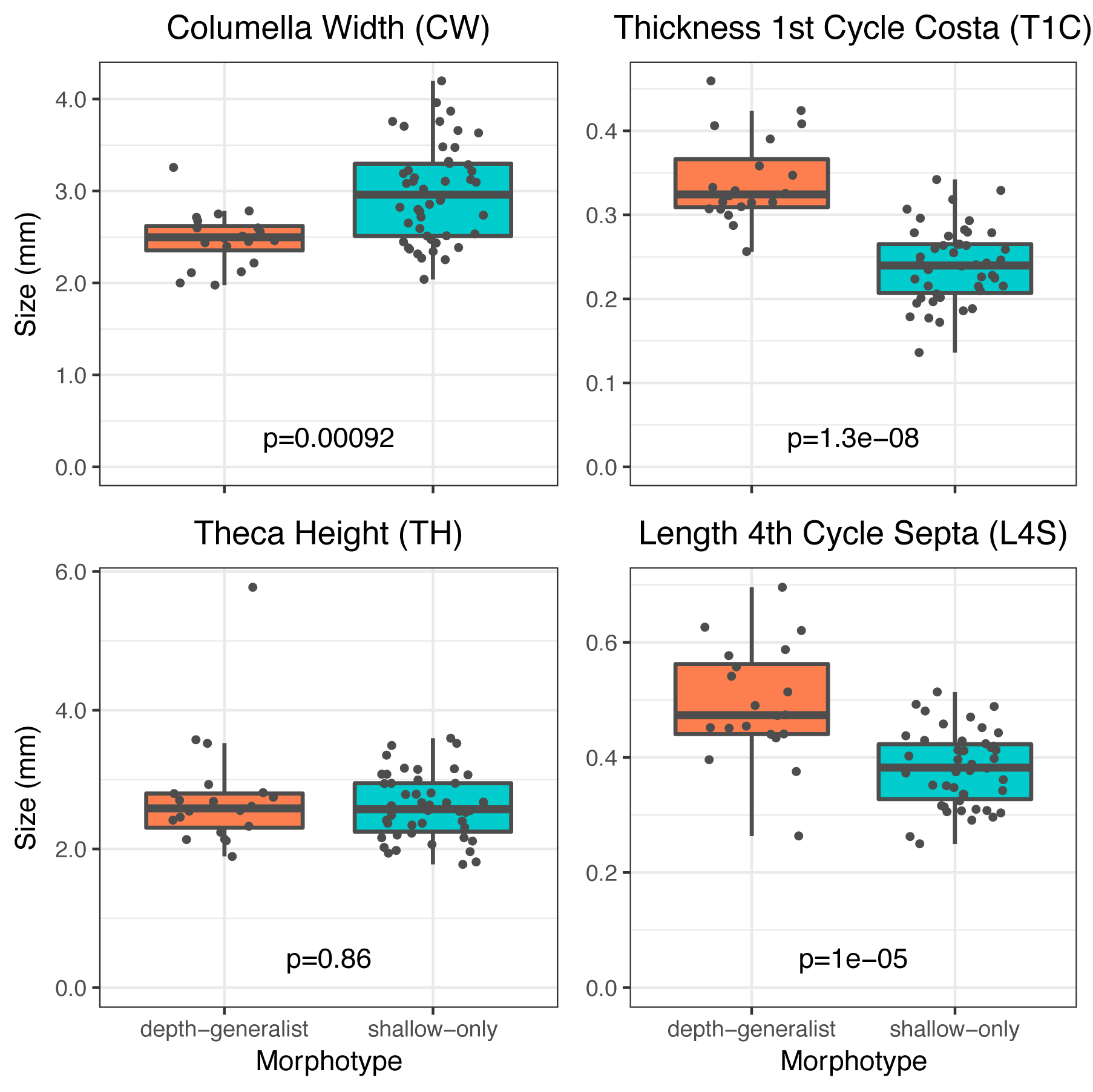

Supplement: S4 Fig — Boxplots with sample overlays for columella width (CW), thickness of the first cycle costa (T1C), theca height (TH), and length of fourth cycle septa (L4S) across depth-generalist (n = 20) and shallow (n = 46) morphotypes sampled within the shallow zone of West and East FGB. Overall p values represent Mann-Whitney U tests between morphotype for each metric. (TIF) [file pone.0203732.s004.tif]

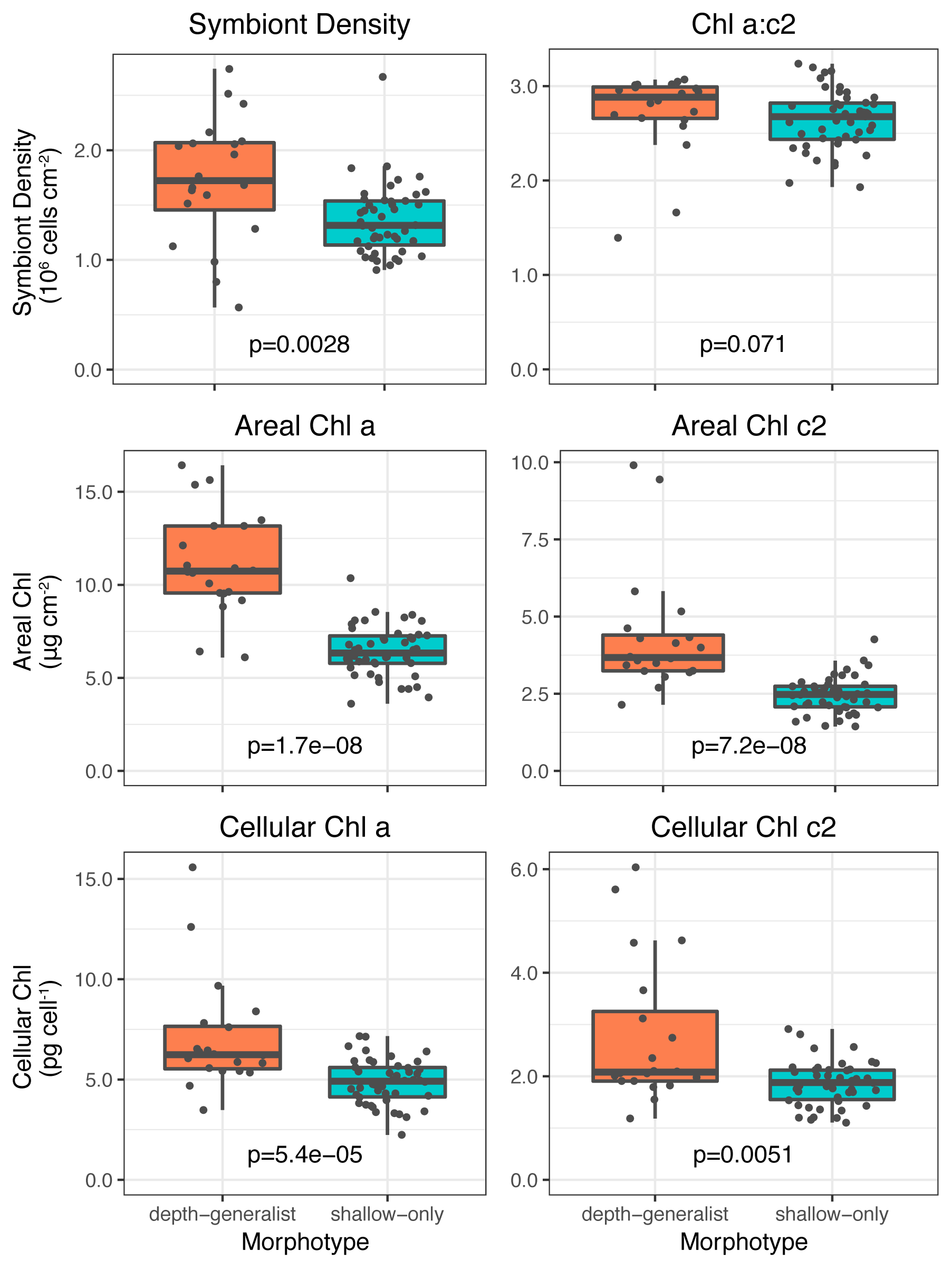

Supplement: S5 Fig — Boxplots with sample overlays for symbiont density, chlorophyll a:c2, areal chlorophyll a, areal chlorophyll c2, cellular chlorophyll a, and cellular chlorophyll c2 across depth-generalist (n = 20) and shallow (n = 46) morphotypes. Overall p values represent Mann-Whitney U tests between morphotype for each metric. (TIF) [file pone.0203732.s005.tif]

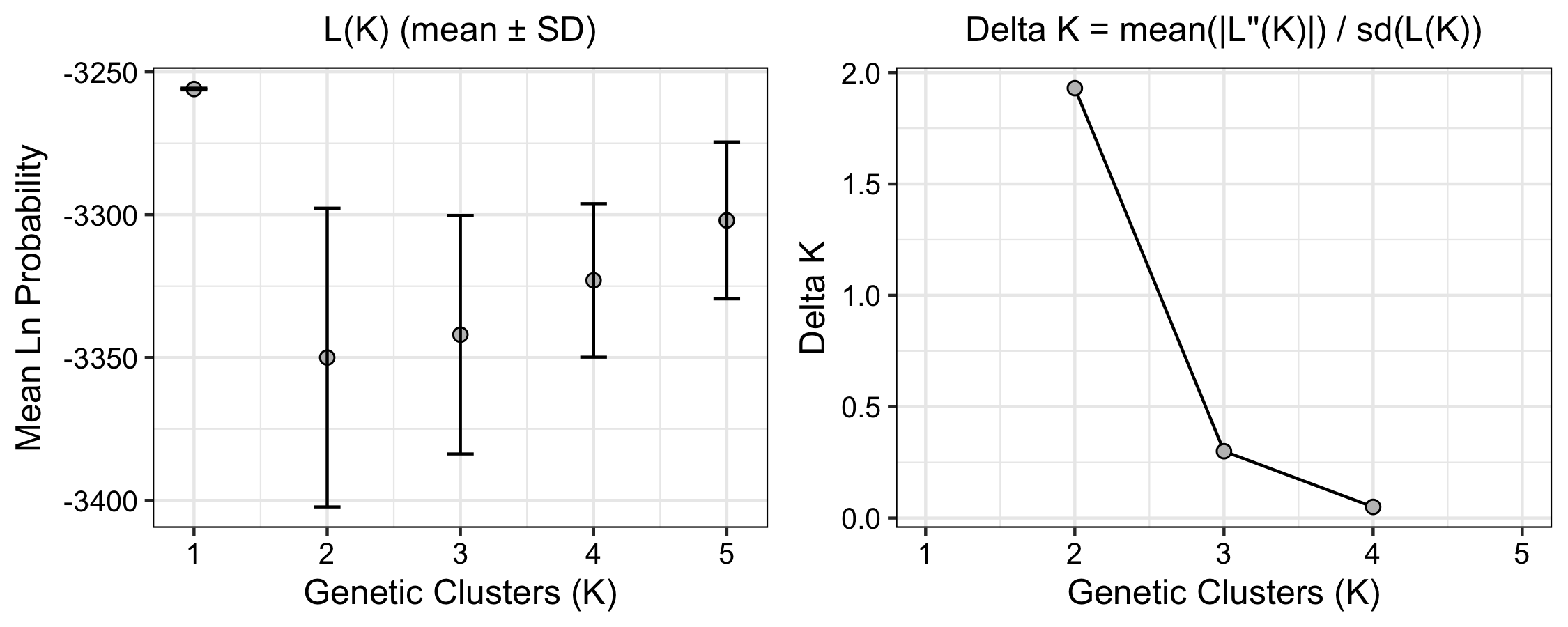

Supplement: S6 Fig — Plots describing the process behind population cluster (K) selection in structure analysis of depth-generalist and shallow populations using the k-means clustering method. Ten replicate structure models were run across a range of K values from 1–5 and model log likelihoods were compared. The Evanno method was used to determine the most likely number of K by identifying the largest change in likelihood (L(K)) and by comparing model probabilities in conjunction with variance (Delta K). Error bars represent standard deviation of the mean. (TIF) [file pone.0203732.s006.tif]
